# On the other end of the line: Extracellular vesicle-mediated communication in glaucoma

**DOI:** 10.3389/fnana.2023.1148956

**Published:** 2023-04-11

**Authors:** Cristiano Lucci, Lies De Groef

**Affiliations:** Cellular Communication and Neurodegeneration Research Group, Department of Biology, Leuven Brain Institute, KU Leuven, Leuven, Belgium

**Keywords:** glaucoma, extracellular vesicles (EVs), biomarkers, retina, miRNAs

## Abstract

In the last decade, extracellular vesicles (EVs) have emerged as a promising field of research due to their ability to participate in cell-to-cell communication *via* the transfer of their very diverse and complex cargo. The latter reflects the nature and physiological state of the cell of origin and, as such, EVs may not only play a pivotal role in the cellular events that culminate into disease, but also hold great potential as drug delivery vehicles and biomarkers. Yet, their role in glaucoma, the leading cause of irreversible blindness worldwide, has not been fully studied. Here, we provide an overview of the different EV subtypes along with their biogenesis and content. We elaborate on how EVs released by different cell types can exert a specific function in the context of glaucoma. Finally, we discuss how these EVs provide opportunities to be used as biomarkers for diagnosis and monitoring of disease.

## 1. Introduction

Glaucoma is the world’s leading cause of irreversible blindness and is characterized by the slow, progressive degeneration of retinal ganglion cells (RGCs) and their axons, with consequent optic nerve atrophy (Tham et al., 2014). RGCs, as all neurons in the central nervous system (CNS), are irreplaceable, making their dysfunction and subsequent loss detrimental to vision and thus, drastically impacting the quality of life of the affected patients. Increased intraocular pressure (IOP) is the major, modifiable risk factor for glaucoma, yet, no neuroprotective therapies currently exist, and many patients continue to lose vision, even with normal eye pressure (Boia et al., 2020). This treatment gap is likely due to the complex etiology of glaucoma, with both genetic and environmental factors involved, emphasizing the need for alternative therapeutic approaches tackling this multifactorial disease.

As part of the CNS, the retina is a complex tissue with various resident cell types, and considerable effort has been devoted to understanding the mechanisms by which these cells communicate with each other and orchestrate retinal homeostasis. In this context, extracellular vesicles (EVs), nanosized membranous vesicles bounded by a lipid bilayer, have emerged as important mediators of this intercellular communication. Released by all cell types, EVs are considered crucial in cell-to-cell communication due to their capacity to transfer bioactive molecules (e.g., nucleic acids, proteins, and lipids) to both surrounding and distant cells, whereby they can impact the phenotype of the recipient cell (Van Niel et al., 2018). Because of this unique characteristic, EVs have great potential as drug delivery vehicles (Liu and Su, 2019). Moreover, as they carry a “liquid biopsy” of the donor cells, and their cargo hence reflects the (patho)physiological state of this cell, EVs have also caught interest as biomarkers for diagnosing diseases (Younas et al., 2022).

In the visual system, EVs released by different cell types are found in several ocular fluids such as aqueous humor, vitreous humor, tears, and blood (Dismuke et al., 2015; Zhao et al., 2018; Rossi et al., 2019; Tamkovich et al., 2019). Even though their roles have been increasingly explored in other retinal diseases such as age-related macular degeneration and diabetic retinopathies, the role of EVs in both the establishment and progression of glaucoma and traumatic optic neuropathies remain largely unknown. In this review, we aim at providing a comprehensive state-of-the-art on the role of EVs in glaucoma pathogenesis. We first delineate an overview of the different EV subtypes along with their biogenesis, content, and isolation techniques currently available. Next, we elaborate on how EVs released by different cell types can exert a specific function in retinal homeostasis and glaucoma. Finally, we focus on how these EVs provide promising opportunities to be used as biomarkers for the disease.

## 2. Overview and biogenesis of EVs

**FIGURE 1:**
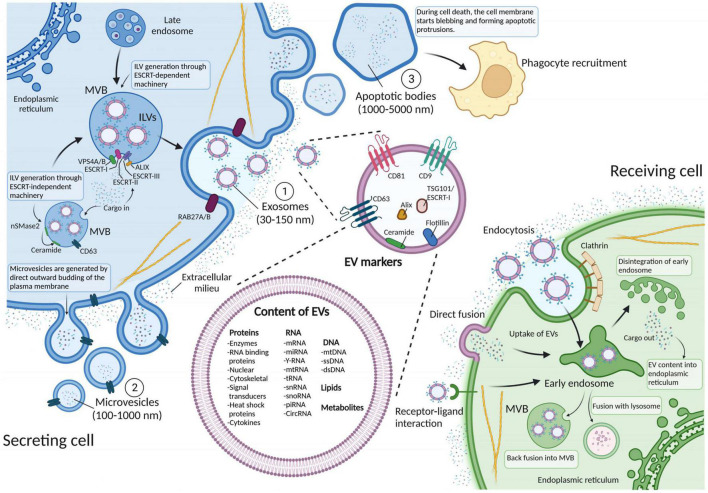
Schematic representation of biogenesis, release, and internalization of extracellular vesicles (EVs), along with markers used for EV identification. The three subtypes of EVs are generated through three different biogenesis pathways. (1) *Exosomes* (30–150 nm) are generated *via* the endosomal pathway through mechanisms that can be either endosomal sorting complex required for transport (ESCRT)-dependent or -independent. These mechanisms orchestrate the invagination of intraluminal vesicles (ILVs) within the multivesicular body (MVB), before being fused with the plasma membrane and released into the extracellular space. (2) *Microvesicles* (100–1,000 nm) are produced when the plasma membrane buds outward, detaches, and becomes a vesicle. (3) *Apoptotic bodies* (1,000–5,000 nm) appear after the disassembly of an apoptotic cell into subcellular fragments and are removed through macrophage recognition of plasma membrane markers followed by phagocytosis. Both exosomes and microvesicles can transfer their cargoes to the recipient cells *via* endocytosis, direct fusion and receptor ligand interaction. EVs that are taken up can then either get degraded by lysosomes or fuse with pre-existing early endosomes and subsequently disintegrate and release their contents into the endoplasmic reticulum or form MVBs again. EV surface proteins include tetraspanins, integrins and immunomodulatory proteins, whereas their cargo can instead contain different types of cell surface proteins, intracellular protein, RNA, DNA, lipids, and metabolites. Figure created with BioRender.com.

Extracellular vesicles are membrane-derived vesicles released by cells into the extracellular space, with a role in cell-to-cell communication and capable of regulating a plethora of biological processes (Valadi et al., 2007; Skog et al., 2008). To date, the consensus is to divide EVs into three subtypes according to the mode of biogenesis and release: *exosomes* (30–150 nm), *microvesicles* (100–1,000 nm), and *apoptotic bodies* (1,000–5,000 nm) (Figure 1). However, novel types of EVs that seem to differ from these three categories have been reported in this rapidly evolving research field, e.g., EVs that are even smaller than exosomes (Zijlstra and Di Vizio, 2018; Lee et al., 2019).

### 2.1. Exosomes

Exosomes are generated through inward invagination of the late endosomal membrane to give rise to the multivesicular body (MVB) (Figure 1). Several proteins are required not only for the invagination *per se* but also for recruitment of the EV cargo: the endosomal sorting complex required for transport (ESCRT) machinery, which is composed of four protein complexes (ESCRT-0, -I, -II, and -III) coupled with accessory proteins (Alix, VPS4, and VTA-1). These proteins sequentially act to recognize and load future exosome cargo into the lumen of endosomes and form intraluminal vesicles (ILVs) (Christ et al., 2017). Recently, it has been proposed an alternative ESCRT-independent mechanism of exosome generation, that involves the spontaneous negative curvature of the MVB membrane to form ILVs through sphingomyelinase hydrolysis and ceramide formation *via* neutral sphingomyelinase 2 (nSmase2) (Verderio et al., 2018). Either way, the MVB then fuses with the cellular membrane and releases the ILVs (now defined as exosomes) into the extracellular space by means of the RAS-related proteins RAB27A and RAB27B (Johnstone et al., 1987; Van Niel et al., 2018).

### 2.2. Microvesicles

Microvesicles are produced when the plasma membrane buds outward and detaches (Figure 1), but their biogenesis is less well characterized compared to exosomes. One possible mechanism requires the recruitment of the same ESCRT machinery that induces the formation of ILVs in the MVB (Lee et al., 2015; Christ et al., 2017). In this way, microvesicles can be generated and pinched off *via* recruitment of the negative curvature-promoting ESCRT-III proteins (Chiaruttini et al., 2015; McCullough et al., 2015). Alternatively, vesicle budding can also occur in response to plasma membrane wounding as a repair mechanism of damaged membranes (Jimenez et al., 2014). Moreover, mechanisms that can produce or alter plasma membrane asymmetry with respect to lipids have also been linked to microvesicles biogenesis (Clark, 2011). This process is mediated by the activity of aminophospholipid translocases (flippases and floppases), scramblases, and calpain, that induce the switching of phospholipids from the outer to the inner leaflet of the plasma membrane (reviewed in Van Niel et al., 2018). As a consequence, any modification in the ceramide content on the outer leaflet *via* acid sphingomyelinase activity can also trigger membrane curvature and induce microvesicle release (Bianco et al., 2009; Awojoodu et al., 2014).

### 2.3. Apoptotic bodies

Apoptotic bodies are formed after the disassembly of an apoptotic cell into subcellular fragments (Figure 1). In fact, after programmed cell death triggered by either a normal physiological response or a pathogenic event, the cell membrane starts blebbing and forming apoptotic protrusions. These protrusions then disassemble to generate apoptotic bodies which can engulf cellular organelles (Hayakawa et al., 2016), nuclear genomic DNA (Bergsmedh et al., 2001) and randomly enclosed cargo (Atkin-Smith et al., 2015). Generally, apoptotic bodies are removed *via* macrophage recognition of plasma membrane markers followed by phagocytosis (Lane et al., 2005).

Despite this classification, each EV subtype does not have specific markers. Hence, the International Society for Extracellular Vesicles (ISEV) has suggested the generic term “EVs” for vesicles naturally released from cells (Théry et al., 2018). In line with this, even though the majority of the research cited in this review utilizes specific nomenclature to describe EV subtypes, we decided to use the general term “EVs.”

## 3. EV content and interaction with cells

Extracellular vesicles are released by virtually all cells under both normal and pathological conditions (Kalargyrou et al., 2023). Thus, the content of each particle, whether released by a healthy, a stressed or diseased cell, or from microorganisms, can differ greatly. EV cargo generally includes nucleic acids, proteins, lipids, enzymes, and metabolites (Figure 1) that reflect the physiological state of the secreting cell (Kalluri and LeBleu, 2020). Deep sequencing studies have demonstrated that EVs can contain many RNA biotypes, including intact mRNA, circRNA and non-coding RNA types such as miRNA, snRNA, lncRNA, vault RNA, Y-RNA, tRNA, and rRNA (Nolte’T Hoen et al., 2012; Jenjaroenpun et al., 2013; Mesquita-Ribeiro et al., 2021). Importantly, the EV-derived RNA content is stable because it is protected from RNases (Cheng et al., 2014) and, as a consequence, it can effectively alter the phenotype of the recipient cell upon uptake (Kalluri and LeBleu, 2020).

Besides genetic material, EVs are also highly enriched in proteins. In fact, because of their biogenesis, exosomes contain enriched endosome-associated components, such as Annexins, flotillins, and tetraspanins (e.g., CD9, CD63, and CD81), (Kowal et al., 2016), whereas microvesicles are typically characterized by lipid composition, plasma membrane receptors and molecules that reflect the cell of origin (Van Niel et al., 2018). Apart from cell-surface proteins and soluble proteins associated with the extracellular milieu, EVs can also contain various intracellular proteins derived from the secreting cell. According to the latest version of the sub-database ExoCarta (January, 2023),^1^ 41,860 proteins, 7,784 RNA entries and 1,116 lipid molecules have been detected within EVs from multiple organisms (Keerthikumar et al., 2016). Moreover, as for nucleic acids, EV proteins are stable (Schey et al., 2015) and can act directly on target cells. As their proteinaceous cargo, as well as nucleic acids such as miRNA, is a mirror of the releasing cell, EVs may provide direct information to diagnose and monitor disease (Fiandaca et al., 2015; Lee et al., 2021). Importantly, EVs are also believed to be involved in the spreading of toxic proteins in neurodegenerative diseases such as Alzheimer’s, Creutzfeldt-Jacob, amyotrophic lateral sclerosis and Parkinson’s disease (reviewed in Janas et al., 2016). In these conditions, a prion-like disease progression has been proposed, and EVs appear to be the major vehicles that shuttle toxic proteins (amyloid, tau, PrP, TDP-43, and α-synuclein) out of the cell and seed protein aggregation in acceptor cells (Fevrier et al., 2004).

Regardless of their content, once released into the extracellular space, EVs may exploit different types of interactions to functionally communicate with the recipient cells. This includes the direct release of EV content in the extracellular space, a receptor-mediated EV binding to the recipient cell surface, EV-plasma membrane fusion, and uptake by endocytosis (Figure 1). These interactions are known to be mediated by molecules such as tetraspanins, integrins, lectins, proteoglycans, lipids, and extracellular matrix (ECM) components. Moreover, ligand-receptor interactions, besides serving as a tool to “hijack” EVs toward specific cells (Hoshino et al., 2015), are believed to be the major responsible for many targeted EV-mediated biological effects (Maas et al., 2017). However, for RNA or cytoplasmic protein delivery, EVs must also release their cargo into the recipient cells. This can be achieved by either direct fusion with the plasma membrane or with the endosomal membrane upon endocytosis *via* clathrin-dependent or clathrin-independent mechanisms (extensively reviewed in Van Niel et al., 2018).

Once inside the cell, it is still unclear how the EV cargo is being released. One possibility is that a fusion between the EVs and endosomal membranes occurs, leading to cargo delivery into the cytoplasm of the recipient cell, prior to degradation *via* lysosomes (Kalluri and LeBleu, 2020).

## 4. EV isolation techniques

**FIGURE 2:**
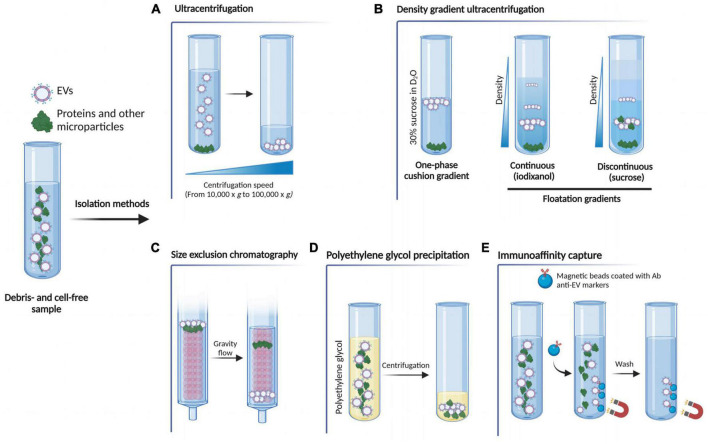
Schematic representation of the most common EV isolation methods. **(A)** Ultracentrifugation exploits high centrifugal forces to separate the EVs from the rest of the sample. **(B)** Density gradient ultracentrifugation separates EVs according to their size, mass and density. The sample can be placed on top of a 30% sucrose in D_2_O solution (one-phase cushion gradients) and, upon ultracentrifugation, denser molecules sediment on the bottom of the tube, whilst the EV fraction stays on top according to its density. In floatation gradients, the sample is placed either at the bottom or at the top of the continuous (iodixanol) or discontinuous (sucrose) gradient and the molecules float up or sediment down to the point where their density is the same as the density of the solution. **(C)** Size exclusion chromatography uses a porous polymer that separates molecules based on size. Proteins are retained by the column by entering the pores of the polymer, so they elute later than EVs. By contrast, EVs, having a bigger size than the pores of the polymer, pass through the column quicker and so elute first. **(D)** Polyethylene glycol (PEG) precipitation exploits an aqueous PEG solution to wrap EVs, producing particle aggregates that can be then pelletted *via* centrifugation. **(E)** Immunoaffinity capture isolation uses the binding between an EV membrane protein marker and its specific antibody to remove other non-specific components. Figure created with BioRender.com.

Isolating EVs from their biological source (i.e., tissue, biofluid, and culture medium) is a critical step when investigating EVs as functional mediators of intercellular communication, drug delivery vehicles, and circulating/systemic biomarkers. In fact, even though diverse EV separation techniques are available based on their different properties (e.g., size, density, and composition), each method comes with its own range of EV purity and yield, that needs to be coupled with quality control measures to confirm the absence of contaminants and the success of the separation *per se* (Théry et al., 2018). It then becomes fundamental to select the technique (or a combination of them Ayala-Mar et al., 2019) that is most suitable for the downstream applications. Here, we outline the most common EV isolation techniques (Figure 2), highlighting their pros and cons.

### 4.1. Ultracentrifugation

Ultracentrifugation is the most widely used and conventional method for EV isolation that exploits the centrifugal force to separate the EVs from the rest of the sample (Théry et al., 2006). Even though there is no standardized protocol yet, this method is based on sequential centrifugations, starting from lower speeds (such as 200 × *g* and 2,000 × *g*) to remove cells, cell debris and apoptotic bodies from the sample, up to 10,000 × *g* to pellet larger microparticles. Finally, two centrifugation steps at 100,000 × *g* are needed to pellet EVs (Figure 2A). Despite its simplicity and low cost, this technique harbors several drawbacks. For example, high centrifugal forces may cause damage to EVs (Nordin et al., 2015; Li et al., 2017), and the final pellet does not exclusively contain EVs, as both protein aggregates and high-density lipoproteins co-precipitate along with the EV particles (Théry et al., 2006; Ramirez et al., 2018). To overcome this issue, additional washing steps after ultracentrifugation can be performed to increase the purity of the pellet, but this can also lead to a lower EV yield (Théry et al., 2006). Moreover, it is a time-consuming and an equipment-dependent method.

### 4.2. Density gradient ultracentrifugation

This method is a variation of the ultracentrifugation described above, in which EVs are separated according to their size, mass and density by exploiting solutions with progressively decreasing densities from the bottom to the top of the tube (Li et al., 2017; Carnino et al., 2019). There are two main types of gradients: one-phase cushion gradients and floatation gradients. In the first type, the EV biological source is placed on the top of a 30% sucrose in D_2_O solution and, upon ultracentrifugation, denser molecules (i.e., protein aggregates) sediment on the bottom of the tube, whilst the EV fraction stays on top according to its density. In the second, the sample is placed either at the bottom or at the top of the continuous (iodixanol) or discontinuous (sucrose) gradient and the molecules float up or sediment down to the point where their density is the same as the density of the solution (Théry et al., 2006; Figure 2B). This technique overcomes the major flaw of the conventional ultracentrifugation, as it increases both the resolution and purity of the separated EV fraction (Van Deun et al., 2014). However, the final EV yield is poor and is a time-consuming and equipment-dependent technique.

### 4.3. Size exclusion chromatography

Extracellular vesicles can be separated according to their size using size exclusion chromatography, a technique that has become increasingly popular, as it allows the separation of EVs with high purity while preserving their biological activity (Nordin et al., 2015; Hong et al., 2016). In this method, molecules and particles pass through a column composed of a porous polymer (matrix or resin), which is the stationary phase of the column, pushed by a mobile phase. Small particles (i.e., proteins) are retained by the column by entering the pores of the polymer, so they elute later than EVs. By contrast, EVs, having a bigger size than the pores of the polymer, pass through the column quicker and so elute first (Grubisic et al., 1967; Figure 2C). In this way, small vesicles are separated from large vesicles, as well as removing non-EV-bound soluble contaminants, such as plasma proteins, urine protein complexes and high-density lipoproteins, resulting in a purer EV preparation when compared to other methods (Böing et al., 2014; Gámez-Valero et al., 2016). However, it is worth noting that this method cannot differentiate between EV subtypes of the same size, and depending on the starting biofluid volume, the final EV yield may be relatively low.

### 4.4. Polyethylene glycol precipitation

This precipitation-based technique exploits an aqueous polyethylene glycol (PEG) solution to wrap EVs, producing particle aggregates that can be then pelletted *via* relatively low-speed centrifugation (reviewed in Stam et al., 2021; Figure 2D). The size range of the EVs separated using this protocol, can be compared to that obtained with other methods but the purity of the EV fraction is largely affected by the presence of co-precipitating non-EV soluble proteins, immunoglobulins and immune complexes (Lobb et al., 2015; Konoshenko et al., 2018). Moreover, the precipitating agent is often retained in the final EV pellet, which may interfere with some downstream analysis such as electron microscopy, and affect the EV biological function (Rider et al., 2016; Martins et al., 2018; McNamara et al., 2019). Overall, because of its simplicity, low cost and easy implementation, this method constitutes a valuable option for the separation of EVs.

### 4.5. Immunoaffinity capture

Immunoaffinity-based protocols are undoubtedly one of the best methods to obtain highly purified and selected EV samples. The key concept behind these methods is the binding between an EV membrane protein marker and a specific antibody. Then, after removing other non-specific components with a series of washes, only the EV fraction containing the target protein is retained (reviewed in McNamara and Dittmer, 2020). This can be achieved by using a microtiter plate-based technique, magnetic beads and affinity columns (Zarovni et al., 2015; Multia et al., 2019; Figure 2E). Nonetheless, since a generic EV surface marker has not been identified yet, the EV separation will be biased by the selected capture antibody, while all the others EVs present in the sample excluded, resulting in a lower yield when compared to other isolation methods (Kowal et al., 2016). For this reason, this methodology is recommended only when a specific EV sub-population needs to be studied.

Extracellular vesicle isolation methods are constantly evolving, and in fact, besides the techniques mentioned above, newly developed approaches or modifications to existing ones are emerging, such as tangential flow filtration (Busatto et al., 2018; Haraszti et al., 2018), field-flow fractionation and microfluidic devices (reviewed in Stam et al., 2021). Thus, the possibility to separate EVs from any biological source is getting closer to reality, even though it is important to note that an EV isolation method compatible with all the biological sources and downstream analysis, is not currently available. For this reason, all the isolations should be well detailed and documented so that both reliability and reproducibility are guaranteed. As it will be mentioned below, this becomes fundamental when EVs derived from different sources (i.e., mesenchymal stem cells-derived EVs) constitute a promising cell-free therapeutical approach. In fact, if not controlled, the selected EV isolation protocol would give rise to different and unconclusive results even though applied to similar experimental designs.

## 5. EVs in glaucoma and traumatic optic neuropathies

**FIGURE 3:**
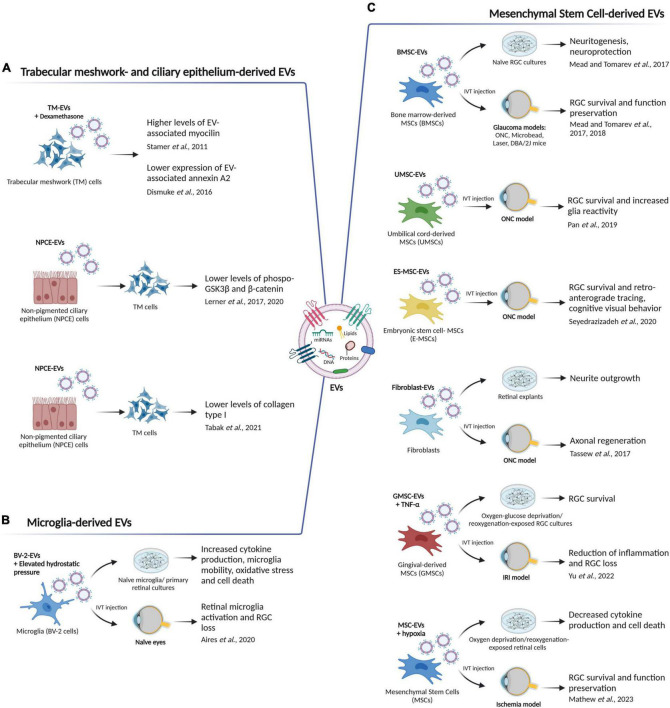
The role of EVs in retinal homeostasis and glaucoma. **(A)** Mesenchymal stem cell-derived EVs (MSC-EVs) have been shown to improve retinal ganglion cell (RGC) survival, promote neurite outgrowth and axon regeneration, and increased glia reactivity in different *in vitro* and *in vivo* glaucoma models. ONC, optic nerve crush; IVT, intravitreal; IRI, ischemia reperfusion injury. **(B)** Trabecular meshwork (TM)- and non-pigmented ciliary epithelium (NPCE)-derived EVs can participate in extracellular matrix (ECM) remodeling by triggering an alteration in EV-associated myocilin and annexin A2 levels, by regulating phospho-GSK3β and β-catenin expression and thus impinging on the Wnt signaling pathway, or by altering the formation and secretion of collagen type I. **(C)** Microglia-derived EVs can act as vehicles/messengers to increase cytokine production, microglia mobility, oxidative stress and retinal cell death *in vitro*, and to induce RGC loss and microglia reactivity *in vivo*. Figure created with BioRender.com.

The intrinsic capacity of EVs to carry bioactive molecules, which are in turn capable of altering the recipient cell phenotype, has been widely recognized. However, the untangling of the molecular mechanisms through which these molecules are impinging on both pathological and physiological cellular processes is instead proceeding at a slow pace. As a consequence, the number of studies attempting to dissect the function of EVs in glaucoma and traumatic optic neuropathies is still limited. Here, we gather EV findings in retinal homeostasis and glaucomatous retinal degeneration (Figure 3). We describe how under pathologic conditions and elevated ocular pressure, EVs originating from exogenous cell populations (e.g., mesenchymal stem cells) or from more autochthonous ocular compartments can have an impact on RGC survival or modulate retinal homeostasis.

### 5.1. Trabecular meshwork- and ciliary epithelium-derived EVs

As mentioned in the introductory section of this review, reducing IOP is the only treatment that has been shown to slow down glaucoma progression in a large subset of glaucoma patients. The trabecular meshwork (TM) is a porous structure located in the anterior chamber of the eye and composed of endothelial cells embedded in a dense extracellular matrix (ECM) (O’Callaghan et al., 2017). Due to its role in regulating the outflow of aqueous humor (AH), the TM is considered a major target to control IOP. Indeed, a higher resistance to aqueous outflow leads to an increased IOP (Vranka et al., 2015), and even though the exact mechanism controlling outflow resistance of the TM is still unknown, ECM remodeling has a key role in maintaining a healthy IOP (Vranka et al., 2015). In fact, a non-physiological accumulation of ECM is a common feature of two major forms of glaucoma: primary open-angle glaucoma and corticosteroid-induced glaucoma (Tektas and Lutjen-Drecoll, 2009).

When the protein expression pattern of human TM cell-derived EVs was examined and compared to that of EVs purified from urine as a control, it showed an enrichment of the glaucoma-associated protein myocilin (Stamer et al., 2011). In addition, treatment of the TM cells with the corticosteroid dexamethasone, triggered an increase in the EV-associated myocilin levels suggesting a functional implication of the EVs in the control of IOP (Stamer et al., 2011). Glaucoma-linked myocilin mutations in fact, may impinge on the exosomal pathway, affecting the ability of TM cells to process cell debris from the AH and resulting in an increased outflow resistance and elevated IOP. Coherently, in a following study, TM-EVs released from dexamethasone-treated TM cells showed changes in the expression of the heparin/heparan sulfate binding protein annexin A2 (Dismuke et al., 2016). This phenotype not only mimics the ECM alterations seen in many glaucoma patients, especially in those with corticosteroid-induced glaucoma, but also reiterates a role of EVs in cell-matrix interactions (Dismuke et al., 2016; Figure 3A).

Extracellular vesicle-mediated intercellular communication is seldomly a unidirectional process, and EVs rather function in reciprocal signaling between cells. In this context, given that non-pigmented ciliary epithelium (NPCE) is the site of AH production, it is not surprising that NPCE cell-derived EVs also participate in ECM remodeling and act as intercellular communicators for the transfer of bioactive molecules from the NPCE to the TM (Lerner et al., 2017, 2020; Tabak et al., 2021). In fact, Lerner et al. (2017) showed a specific accumulation of the NPCE-derived EVs in TM cells, which was coupled with changes in Wnt signaling in the recipient cells, as demonstrated by the alterations in both phospho-GSK3β and β-catenin levels. Interestingly, even though the exact underlying mechanism is still unknown, the Wnt signaling pathway has been implicated in both primary open-angle glaucoma (Wang et al., 2008) and TM AH drainage resistance (Villarreal et al., 2014). Later on, the same group validated these findings in a primary cell culture model, and as above, uptake of EVs released by primary NPCE cells led to lower protein levels of both phospho-GSK3β and β-catenin in TM cells (Lerner et al., 2020). Moreover, miRNAome and proteome profiling of NPCE EVs revealed 584 miRNAs and 182 proteins involved in the regulation of TM cellular processes, including cell adhesion, extracellular matrix deposition and Wnt signaling, providing an insight in the physiological role of NPCE EVs (Lerner et al., 2020). Finally, application of NPCE-derived EVs to human TM cells affected the formation and secretion of collagen type I to the ECM (Tabak et al., 2021), suggesting that these EVs can be used to regulate collagen type I fibrillogenesis in TM cells, an important process in ECM remodeling (Tabak et al., 2021). Mechanistically, Wnt signaling converges on the TGF-β pathway to regulate ECM gene expression and plays a role in ECM assembly (Tan et al., 2014). Thus, the aforementioned studies place NPCE- and TM-derived EVs at a signaling crossroad able to impinge on ECM remodeling and IOP regulation, making them a valuable therapeutic target to halt glaucoma pathogenesis (Figure 3A).

### 5.2. Microglia-derived EVs

Considering the cell heterogeneity populating the retina, and that EVs are being released by virtually all cell types, it is not surprising that an ever-growing list of EV populations with different retinal cell origin, is being linked not only to both the onset and progression of glaucomatous retinal degeneration (Aires and Santiago, 2021), but also to other retinal diseases (Shah et al., 2018; Oltra et al., 2019; Saada et al., 2022). In fact, it has been reported that EVs released by a murine microglia cell line (BV-2 cells) upon exposure to elevated hydrostatic pressure-as a model to mimic elevated IOP–(referred to as EHP-BV-EVs) increased the production of pro-inflammatory cytokines (TNF and IL-1β), enhanced microglia motility, expression of major histocompatibility complex class II (MHC-II) molecules and phagocytic efficiency in naïve microglia (Aires et al., 2020). This phenotype was coupled to an increased cell death and reactive oxygen species production in primary retinal neural cell cultures. Moreover, intravitreal injections of EHP-BV-EVs triggered retinal microglia activation and RGC loss *in vivo* (Aires et al., 2020; Figure 3B). It is not clear whether the BV-EV-mediated biological response is caused by their direct action on retinal microglia or if other retinal cells have a contributing effect. However, as speculated by the authors, due to their *in vitro* evidence, it is plausible to think that BV-EVs are at least partially directly impinging on retinal microglia homeostasis (Aires et al., 2020). In general, microglial reactivity plays a dual role in glaucoma progression, being beneficial or detrimental depending on the degree and timing of activation (reviewed in García-Bermúdez et al., 2021). As a consequence, microglia-derived EVs may reflect this duality and thus, differently modulate retinal homeostasis. A better understanding of their role in glaucoma, coupled with a deeper characterization of their content and of the effect they exert on the recipient cells, may pave the way for new therapeutic approaches. Overall, this study indicates that microglia-derived EVs may have a crucial role in glaucoma pathogenesis by acting as vehicles for spreading neuroinflammatory stimuli in the retina.

### 5.3. Mesenchymal stem cell-derived EVs

Extracellular vesicles derived from mesenchymal stem cells (MSCs) have been investigated extensively in many different fields because of their pro-regenerative potential (reviewed in Gowen et al., 2020). The first evidence for a beneficial effect of MSC-derived EVs in glaucoma and traumatic optic neuropathies comes from a study in which EVs isolated from human bone marrow-derived MSCs (BMSC-EVs) were tested both *in vitro* and *in vivo* by using primary RGC cultures and a rat optic nerve crush (ONC) model, respectively, (Mead and Tomarev, 2017). Specifically, treatment of RGC cultures with BMSC-EVs promoted both neuroprotection and neuritogenesis. In addition, intravitreal injection (IVT) of these BMSC-EVs in rats that underwent ONC, preserved retinal function and structure, as measured by electroretinography and optical coherence tomography. BMSC-EVs neuroprotective function was ascertained to be due to miRNAs, since the knockdown of Argonaute-2 (*Ago2*) in BMSCs impaired their therapeutic efficacy (Mead and Tomarev, 2017). As a follow-up study aimed to test the therapeutic potential of BMSC-EVs in more clinically relevant settings, the same authors injected BMSC-EVs into the vitreous of three different rodent glaucoma models: laser- and microbead-induced ocular hypertension models in rat (Mead et al., 2018b), and a genetic DBA/2J mouse model (Mead et al., 2018a). In all the aforementioned models, BMSC-EVs produced RGC survival and preserved their function. Once again, their mechanism of action was linked to the miRNA cargo they delivered into RGCs, demonstrated by *Ago2* knockdown (Mead et al., 2018b). Moreover, in an effort to pinpoint specific miRNAs involved in this BMSC-EV-mediated therapeutic effect, Mead et al. (2018b) detected *via* RNAseq 43 miRNAs that were up-regulated in BMSC-EVs compared to fibroblast-derived EVs. These miRNAs were predicted to impinge on intracellular pathways such as EGF, PDGF, and PTEN signaling; however, functional studies are needed to confirm this interaction. Furthermore, EVs derived from umbilical cord mesenchymal stem cells (UMSCs) and human embryonic mesenchymal stem cells (E-MSCs) have shown similar neuroprotective effects on RGCs in a rodent model of optic nerve injury (Pan et al., 2019; Seyedrazizadeh et al., 2020). As well, the application of fibroblast-derived EVs shortly after optic nerve injury can promote axonal regeneration by activating the autocrine Wnt10b-mTOR pathway (Tassew et al., 2017). This seems to be in contrast with previous findings from Mead et al. (2018b) in which fibroblast derived-EVs did not display a neuroprotective activity. However, this discrepancy could be possibly due to different EV isolation procedures that led to different EV cargoes and thus to a different axogenic effect. In the context of retinal ischemia-reperfusion injury (IRI), which is one of the main pathogenic mechanisms of glaucoma (Vidal-Sanz et al., 2001; Sun et al., 2010; Rovere et al., 2016; Wang et al., 2017), IVT injections of TNF-α-stimulated gingival MSC-EVs into mice with IRI reduced inflammation and RGC loss (Yu et al., 2022). Similar results were also obtained *in vitro*, as RGCs cultures that underwent oxygen-glucose deprivation/reoxygenation showed higher survival compared to the control condition upon application of gingival MSC-EVs. Specifically, this neuroprotective function was mediated by miR-21-5p *via* the targeting of programmed cell death 4 (PDCD4) (Yu et al., 2022). Lastly, EVs derived from human MSCs were able to ameliorate both neuroinflammation and apoptosis in the retina, and enhanced functional recovery in a rat model of retinal ischemia (Mathew et al., 2019). Interestingly, the administration of these EVs also triggered mild toxicity in the RGC layer of the non-ischemic condition (Mathew et al., 2019). Thus, in a follow-up study aimed at enhancing the MSC-EV neuroprotective and anti-inflammatory properties, the same authors demonstrated that hypoxic preconditioning of the MSCs generated EVs that, once IVT injected in a rat model of retinal ischemia, not only restored retinal function but also prevented loss of RGCs (Mathew et al., 2023). Moreover, the MSC-EVs activity was also tested in an *in vitro* hypoxia model of retinal cells, where these nanoparticles reduced inflammatory cytokine production in microglia and lowered oxygen free radicals in Müller glia and microvascular endothelial cells *via* miR-424-5p (Mathew et al., 2023).

The studies above have altogether demonstrated the therapeutic potential of MSC-EVs in preclinical models of both glaucoma and traumatic optic neuropathies (Figure 3C), however their mechanism of action is still unclear. In fact, while a direct neuroprotective effect of MSC-EVs on RGCs has been speculated (Mead et al., 2018b), an indirect effect through glial intermediaries or immune modulation cannot be excluded (Mead and Tomarev, 2017; Pan et al., 2019; Seyedrazizadeh et al., 2020; Yu et al., 2022). Indeed, also glial and immune cells may be targeted by MSC-EVs, and therefore a therapeutic effect may be achieved by modulating neuron-glia or neuro-immune cross-talk. While further work is necessary to pinpoint specific MSC-EV content and targets, overall, these studies represent an encouraging proof-of-concept that MSC-EVs constitute a valuable cell-free alternative to MSC-based therapies for the protection of RGCs in a cross-species manner.

## 6. EVs as potential biomarkers for glaucoma

Besides their function as mediators of cell-to-cell communication, EVs have also been investigated as circulating/systemic biomarkers. In fact, as their cargo reflects the nature and physiological state of their cell of origin, the EV molecular content (i.e., miRNAs, inflammatory proteins) can be characterized to generate a profile, which in turn can be exploited to detect, monitor and prognosticate disease. However, the isolation of ocular EVs is challenging due to the limited volume of the aqueous humor, vitreous humor, and tear fluid. As a consequence, only a few studies have reported the isolation of ocular EVs for the identification of biomarkers for glaucoma.

As described in a previous section of this review, lowering IOP can be achieved by either decreasing the rate of AH production by the ciliary epithelium or by regulating the AH outflow. As AH also constitutes a source of EVs (Perkumas et al., 2007) mainly derived from TM and NPCE cells, it is not surprising that studies aimed at detecting biomarkers for the disease focused on this specific biofluid. In this context, Dismuke et al. (2015) characterized the miRNA content of EVs isolated from human AH collected during cataract surgery. Specifically, more than 10 different miRNAs were identified. Among these, the most abundant species included miR-486-5p, miR-184 and miR-204, the function of which has been indirectly linked to processes important for proper outflow facility regulation (Dismuke et al., 2015). Even though further investigations are necessary to confirm the direct involvement of these miRNAs in these processes, the authors speculated that changes in the AH-EV miRNAs could be used as a biomarker for prognosis and diagnosis of ocular disease (Dismuke et al., 2015). Recently, an exploratory study investigated the size distribution and quantified EVs in AH from patients with pseudoexfoliation glaucoma (An et al., 2022). Even though this work did not address any specific type of EV cargo, the particle count was found to be significantly higher in pseudoexfoliation glaucoma patients when compared to the control group, suggesting a possible EV implication in the pathogenesis of the disease (An et al., 2022). Finally, *in vitro* miRNA gene chip analysis on RNA extracted from human TM-derived EVs revealed that 23 miRNAs were upregulated and three miRNAs downregulated upon transforming growth factor-β2 (TGF-β2) stimulation compared to control conditions (Zhang and Wang, 2021). TGF-β2 can induce ECM remodeling, which in turn, is tightly linked to primary open-angle glaucoma (Fleenor et al., 2006; Tan et al., 2014). For this reason, the aforementioned miRNA signature could constitute potential biomarkers for early primary open-angle glaucoma diagnosis and treatment (Zhang and Wang, 2021).

Extracellular vesicles were also found to be a constitutive component of the vitreous under both physiological and pathological conditions (Zhao et al., 2018). Interestingly, the list of proteins identified in the vitreous-derived EVs included the glaucoma-associated protein myocilin (Zhao et al., 2018). Nonetheless, even though their origin has not been fully elucidated, the presence of retinal proteins in vitreous-derived EVs demonstrates the existence of a communication line between ocular tissues. Recently, in a miRNA microarray analysis performed on CD63+ EVs isolated from the vitreous of a rat model of non-arteritic anterior ischemic optic neuropathy, 38 differently expressed miRNAs were identified (Chen et al., 2023). Among these, the most abundant species included M1/M2 macrophage-related miRNAs (i.e., miR-125a-5p, miR-31a-5p, miR-124-3, miR-182, and miR-181a-5p). Intriguingly, the intravitreal injection of miR-124-3 in rats with the same pathological model, was able to rescue RGC survival when compared to the control group (Chen et al., 2023).

In this context, despite the huge potential of exploiting tear fluid as a non-invasive source of EV biomarkers for retinal diseases, including glaucoma, the number of studies that attempted at investigating EVs present in this biofluid is extremely limited. To our knowledge, Tamkovich et al. (2019) were the first to characterize the EV content of tears obtained from primary open-angle glaucoma patients. Specifically, when compared to those from healthy donors, patient tear-derived EVs contained high concentrations of genomic dsDNA and three differently expressed miRNAs (i.e., miR-146b, miR-16, and miR-126) (Tamkovich et al., 2019). Almost in parallel, a pro-inflammatory protein cargo was also detected in tear-derived EVs obtained from primary open-angle glaucoma patients (Rossi et al., 2019). Specifically, by exploiting an upstream and downstream regulator analysis using Ingenuity Pathway Analysis software on the proteins identified in patients tear-derived EVs, the authors linked the EV-related inflammatory response to a significant increase in the recruitment of neutrophils (Rossi et al., 2019). While further work is required to functionally validate these findings, overall, the above-described studies have confirmed the particularly appealing capacity of tear fluid to be used as source of EV biomarkers for glaucoma.

As for the tears, the possibility to collect blood fractions such as plasma and serum constitutes an equally promising tool for the identification of EV biomarkers in eye diseases, coupled with the bigger volume that can be obtained with this approach compared to other ocular fluids. As consequence, mounting evidence is providing the launching pad for the identification of eye disease-specific biomarkers in the systemic circulation (Mazzeo et al., 2018; Elbay et al., 2019); however, glaucoma-specific biomarkers in blood are still lacking.

## 7. EVs as therapeutic agents

As pointed out throughout this manuscript, EVs are considered important mediators of both biological and pathological processes-not only in eye diseases (Zhang et al., 2021), but also in other contexts (Kalluri and LeBleu, 2020). For this reason, EVs are being actively explored as natural drug delivery systems (Kooijmans et al., 2021), and several features place them as powerful shuttles for the delivery of therapeutic agents. In fact, EVs can overcome the blood-retina/brain barrier (Elliott and He, 2021), and are stable in body fluids. Compared to liposomes or other carriers, EVs can be efficiently taken up by other cells and release a functional cargo with minimal immunogenic reaction upon exogenous administration (Liao et al., 2019). They can be loaded with a specific molecule(s) or compound, yet they also appear to have therapeutic effects *per se*. Indeed, e.g., MSC-derived EVs not only they do not induce toxicity when repeatedly injected both in mice (Mendt et al., 2018) and humans (Kordelas et al., 2014), but they also have a therapeutic effect by themselves (Yeo et al., 2013). Apart from the above described roles of MSC-derived EVs in glaucoma and traumatic optic neuropathies, where they have been used in a cross-species manner, this population of EVs may also have other potential applications in the eye, such as improving retinal laser injury (Yu et al., 2016) and in experimental autoimmune uveoretinitis (Bai et al., 2018). Nonetheless, as will be better discussed in the next section of this review, exploiting EVs as therapeutic agents also presents some drawbacks that need to be overcome.

## 8. Discussion

Because of its complex etiology and multifactorial nature, there is an emerging belief that a future neuroprotective therapy for glaucoma and traumatic optic neuropathies should be based on a “polypharmacological approach” (Weinreb et al., 2014). As already discussed in this review, EVs are natural vehicles for the transfer of their “expensive” cargo, and the recipient cells naturally possess all the tools required to take up EVs (Van Niel et al., 2018; Kalluri and LeBleu, 2020). Thus, EVs are likely to deliver a multi-pronged response to the target cell *via* their complex and diverse cargo, and they hold the potential of being powerful shuttles for the delivery of therapeutic agents to the retina.

In this review, we have focused on the role of EVs in glaucomatous and traumatic optic neuropathies and discussed how EVs regulate cellular biologic functions in different parts of the eye *via* the release of bioactive molecules. We also highlighted the possibility to use them as biomarkers, as well as the advantages of exploiting them as therapeutic agents or drug delivery vehicles. However, we are still far from their successful implementation into clinical therapies. The relatively low EV yield that can be obtained with the present isolation methods, as well as the presence of co-purified contaminants, constitutes a first obstacle, and the large-scale production of clinical-grade EV remains a challenge (Yamashita et al., 2018). Secondly, while no evidence exists for any complications upon administration of EVs into the eye, further toxicology studies are still needed along with more insights into both dosing and biodistribution. Indeed, the biological activity of EVs could be dampened by a rapid clearance and low accumulation in target tissues and cells (Wiklander et al., 2015). Specifically in the eye, IVT injected fluorescently-labeled EVs were cleared from the vitreous, yet were retained in the RGCs *in vivo*, with a retention time between 6 and 14 days (Mathew et al., 2021). A possible approach to increase the retention time of the IVT injected EVs, could be to incorporate the EVs in hydrogel implants which, by acting as reservoirs in the vitreous humor, may ensure a prolonged EV release and an increased therapeutic window with a single injection into the eye (Ilochonwu et al., 2020; Ottonelli et al., 2022). Finally, it is still unclear which EV cargo component is responsible for the therapeutic effects observed in the above-mentioned studies, and many of them do not explore the precise cellular target. This becomes particularly important when exploring their miRNA cargo. miRNAs often converge on a functional outcome by acting on multiple targets belonging to the same or related pathways (Sundermeier and Palczewski, 2016). For this reason, they can be considered intrinsically a multifactorial treatment impinging on the recipient cell phenotype. However, all the miRNA regulatory networks mentioned in this review have been predicted rather than validated. If their mechanism of action can be narrowed down to just a few selected miRNAs/proteins, a potential EV-derived miRNA therapeutic can be further simplified by using these specific candidates. In traumatic optic neuropathies, EV function seems to be mainly mediated by miRNAs and alterations in their cargo during disease are mostly alterations in miRNA (Mead and Tomarev, 2017; Mead et al., 2018b). Nonetheless, the miRNA landscape of RGCs and that of other retinal cells has not been characterized yet. Even if challenging, it would be even more valuable to define the retinal miRNAome changes before and upon injury so that more tailored EV-based therapies can be developed. In eye diseases, IVT administration of compounds is commonly used to treat RGCs. It has been postulated that engineered EV-liposome hybrid nanoparticles, which are in fact synthetic EVs, could be efficient drug delivery vehicles (Gokita et al., 2020; Kiaie et al., 2022). Moreover, much technological progress is being made in engineering EVs (hybrids) to obtain cell type specific delivery of their content (Elkhoury et al., 2022; Evers et al., 2022). The encapsulation of miRNA in EV hybrids can also provide a solution for miRNA-based therapeutic drawbacks–including delivery issues and poor stability of naked miRNA *in vivo*.

Next to their potential as drug delivery tool, EVs also receive a lot of attention in biomarker research. Significant progress has been made in the isolation and analysis of EVs from body fluids to investigate their use as biomarkers for predicting, monitoring and prognosticating disease. While this is an active research field for many neurodegenerative (e.g., Alzheimer’s and Parkinson’s disease), no validated biomarker profile is available for glaucoma yet. Ideally, these biomarkers should be retrieved from biofluids that can be easily accessed, and both aqueous humor and tear fluids constitute two promising routes to achieve this aim. However, the relatively small volumes that can be retrieved, may represent a drawback for the isolation and characterization of EVs. On the other hand, circulating EVs have shown an ability to package proteins and miRNAs involved in retinal disease progression (Mazzeo et al., 2018; Elbay et al., 2019). A potential drawback of using blood as source of eye-derived EVs is that they likely constitute only a small fraction of the total particle count that can be found in this biofluid. It then becomes imperative to implement a comprehensive characterization of ocular cell-derived EVs, before and upon injury/disease to better pinpoint specific glaucoma-linked EV markers and remove potential confounding factors present in the systemic circulation. Finally, a potential EVs source and still largely unexplored alternative in the context of glaucoma, is the cerebrospinal fluid (CSF). In fact, even though its role in optic nerve homeostasis is poorly understood, as it surrounds the nerve within the subarachnoid space, EVs may also round off communication between the CSF and the optic nerve itself (Tsakiri et al., 2015; Mathieu et al., 2017). Moreover, given that both CSF pressure (Berdahl et al., 2008; Ren et al., 2010; Zhang et al., 2015) and flow dynamics (Wostyn et al., 2015) have been implicated in glaucoma pathogenesis, the EVs enclosed within the eye-optic nerve-CSF axis-both as biomarkers and functional mediators-constitute a promising new line of communication that should be further investigated.

Even though still in its infancy, EV biomarker research is a promising field which has the potential to be valuable to patients in the future. All in all, EVs are just starting to unveil the versatility of their cargo and the multitude of processes that they are involved in. Yet, it is clear that their content and role in cell-to-cell signaling adds a whole new layer of complexity to the (patho)physiology of the retina and that we must further explore their role in disease processes, therapeutic approaches and biomarker discovery for glaucoma.

## Author contributions

CL contributed to the conceptualization and writing—original draft. LD contributed to the conceptualization and writing—review and editing. Both authors contributed to the article and approved the submitted version.

## Footnotes

1
http://www.exocarta.org
